# Unveiling the hidden link: elevated platelets and T cell subsets in 5% of moderate COVID-19 patients 48 days post-onset

**DOI:** 10.3389/fcimb.2024.1498491

**Published:** 2024-11-19

**Authors:** Hui An, Ting Li, Lanting Yang, Xinyue Zhang, Hao Hu, Chen Zhang, Yongyu Wang, Shengwei Jin, Ming Li

**Affiliations:** ^1^ Department of Anesthesia and Critical Care, The Second Affiliated Hospital and Yuying Children’s Hospital of Wenzhou Medical University, Wenzhou, Zhejiang, China; ^2^ School of Basic Medical Science, Wenzhou Medical University, Wenzhou, Zhejiang, China; ^3^ Key Laboratory of Pediatric Anesthesiology, Ministry of Education, Wenzhou Medical University, Wenzhou, Zhejiang, China

**Keywords:** COVID-19, long COVID, platelet, T cell subsets, immune homeostasis

## Abstract

**Introduction:**

Platelets are hyperactived during acute COVID-19, promoting clotting and modulating immune-cell responses. Immune thrombocytopenia in adults can manifest as an uncommon complication resulting from various viral infections or as a rare adverse event associated with vaccination. However, their role in convalescent COVID-19 patients remains underexplored. This study examines platelet dynamics early in the pandemic, 48 days post-symptom onset, in unvaccinated patients.

**Methods:**

This longitudinal study included 298 unvaccinated COVID-19 patients (17 mild, 281 moderate) from multiple centers. Clinical evaluations and peripheral lymphocyte subset analyses via flow cytometry were conducted upon admission and on day 48 post-symptom onset (DPSO 48).

**Results:**

At DPSO 48, 5.3% of moderate COVID-19 patients exhibited high platelet counts (>300×10^9^/L), associated with elevated total T-cells (26.4%), CD4 T-cells (24.4%), CD8 T-cells (36.9%), and Tregs (33.9%) compared to patients with normal platelet counts. However, the CD4/CD8 T-cell ratio and T-cell subset frequencies remained unaffected, indicating ongoing T-cell homeostasis restoration. Additionally, a significant positive correlation (r=0.636, p=0.03) was found between platelet counts and B cells in patients with elevated platelet counts.

**Discussion:**

Platelets may play a pivotal role in immune regulation during the recovery phase of COVID-19. Targeting platelets and their secreted mediators could improve immune balance in patients with immune disorders, highlighting a potential therapeutic approach for enhancing recovery in post-COVID-19 patients.

## Introduction

Platelets have a complex impact on the outcome of viral infections, with both favorable and unfavorable effects. Being anucleate cells, platelets contribute to the host defense against viruses by engulfing RNA viruses and recruiting immune cells to eliminate viruses (Negrotto et al., 2015; Zhao et al., 2023), which helps prevent the spread of the viruses. For example, platelets containing SARS-CoV-2 mRNA were detected in COVID-19 patients (Manne et al., 2020; Zaid et al., 2020). Platelets can also assist in regulating immune-cell responses to pathogens by binding and interacting to various leukocytes, such as monocytes, neutrophils, eosinophils, and lymphocytes (Koupenova et al., 2018). In terms of adaptive immune defense, platelets stimulate B cells to produce immunoglobulins (IgG1, IgG2, and IgG3) (Negrotto et al., 2015; Koupenova et al., 2018), thus enhancing the antibody-mediated immune response against the virus. However, platelets can also exacerbate inflammation by increasing the recruitment of neutrophils, promoting the formation of neutrophil extracellular traps (Koupenova et al., 2018), and contributing to an elevated risk of thrombosis (Koupenova et al., 2014; Sciaudone et al., 2023), and tissue injury (Antoniak and Mackman, 2014) during viral infections.

There is evidence to suggest a correlation between platelet abnormalities and COVID-19. Several studies have indicated that patients with COVID-19 demonstrate hyperactive platelets, which are believed to play a role in the pathophysiology of the disease (Chan and Rout, 2020; Manne et al., 2020; Qu et al., 2020; Zaid et al., 2020). In addition, SARS-CoV-2 vaccination can, albeit rarely, induce platelet related vaccine-induced immune thrombotic thrombocytopenia or thrombosis with thrombocytopenia syndrome (Marcucci and Marietta, 2021), highlighting the fundamental pathological condition/disease of platelet disorders in COVID-19.

Epidemiological studies on post-COVID-19 sequelae (also known as long COVID, LC) face considerable challenges in interpretation and integration due to significant disparities in sampling methods, inclusion criteria, participant demographics, diagnostic standards, and overall study designs. The clinical presentations of long COVID are varied and can impact multiple organ systems, with symptoms that may change over time. Current estimates indicate a conservative incidence rate of 10% among infected individuals worldwide; however, the actual number is likely considerably higher due to many unreported cases. Incidence rates for long COVID are estimated to range from 10% to 30% among non-hospitalized patients, 50% to 70% among those who were hospitalized, and 10% to 12% among vaccinated individuals (Davis et al., 2023; Greenhalgh et al., 2024). These symptoms of LC may potentially be linked, at least partially, to immune cell dysregulation (Altmann et al., 2023). The underlying mechanisms of LC are still under investigation. Intriguingly, platelet dysfunction and hypercoagulability have been consistently linked with LC (Pretorius et al., 2021). Platelet-secreted serotonin (5-HT) is known to modulate the function and proliferation of T cells and B cells (Aune et al., 1993; Iken et al., 1995; Abdouh et al., 2001). Recently, several mechanisms have been reported to contribute to the decrease in circulating 5-HT levels in LC patients. These include impaired absorption of tryptophan, the precursor of 5-HT, in the gastrointestinal tract, decreased storage of 5-HT in platelets due to thrombocytopenia, and increased breakdown of 5-HT by enzymes involved in its metabolism (Wong et al., 2023).

Insufficient research has explored the role of platelets in the alterations observed in immune cell profiles among patients recovering from COVID-19. This study addresses this gap by investigating a subgroup of moderate COVID-19 unvaccinated patients (5.3%) who exhibited elevated platelet counts (> 300×10^9^/L), exceeding the upper threshold (above high limited, AHL) on the 48th day post-symptom onset (DPSO 48). The primary objective was to examine the correlation between platelets and various immune cell populations, including CD3^−^CD19^+^ B-cells, different T-cell subsets (CD3^+^CD4^+^ T-cells, CD3^+^CD8^+^ T-cells, double-negative T-cells (DN T-cells), regulatory T-cells (Tregs)), and CD3^−^CD16^+^CD56^+^ natural killer (NK) cells, within the context of moderate COVID-19 cases exhibiting AHL platelet levels and normal platelet levels during recovery.

## Methods

### Ethics statement

This study has obtained ethical approval from the Ethics Committee of Wenzhou Medical University (Ref 2020002). Our research adheres strictly to the ethical guidelines outlined in the 1975 Declaration of Helsinki. Moreover, all participants have provided written informed consent for the collection of samples.

### Study design and participants

**Figure 1:**
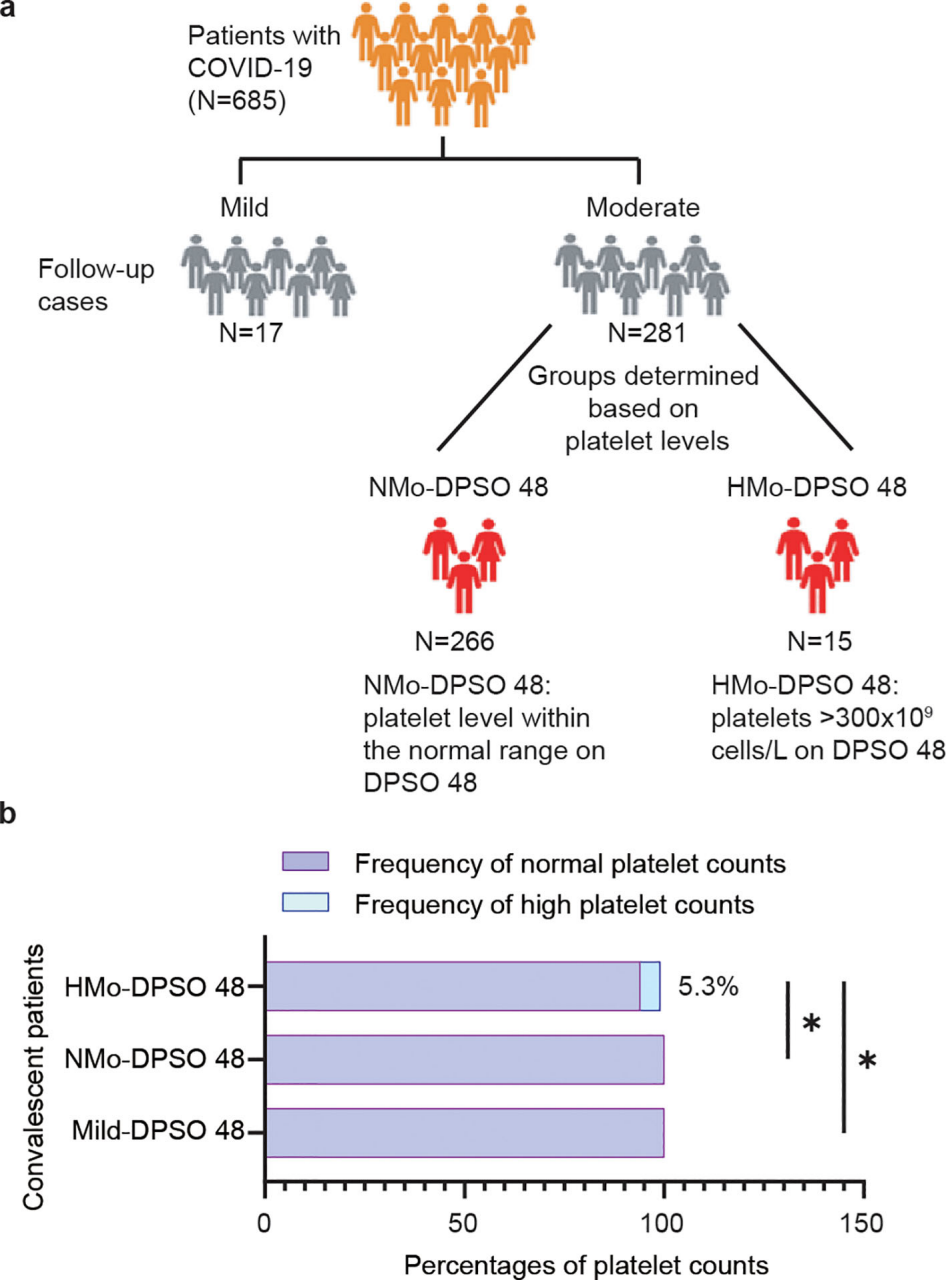
Trial profile. **(A)** Flowchart showing the number of patients enrolled in the study. **(B)** The proportion (%) of cases with a high platelet count (>300×10^9^ cells/L) in the mild and moderate groups at DPSO 48. DPSO 48, 48 Days post-symptom onset. P values were calculated using Fisher exact tests. Statistical significance is denoted by *p<0.05.

In this longitudinal study, 298 cases who had previously recovered from SARS-CoV-2 infection and had not been vaccinated were re-enrolled after providing consent for the follow-up study. These patients constituted a subset of a larger cohort consisting of 685 individuals who were diagnosed with COVID-19 and were admitted to 12 hospitals in Wenzhou City, Zhejiang Province, China between January 17, 2020, and March 20, 2020. Upon admission, the categorization of mild and moderate cases was based on specific criteria. Mild cases were defined as patients displaying mild symptoms but no abnormalities on chest CT scan. On the other hand, moderate cases were characterized by the presence of fever, respiratory tract symptoms, and visible pneumonia on chest CT scan. Discharged COVID-19 patients were required to fulfil specific criteria, which included being fever-free and having the resolution of respiratory symptoms for a minimum of 3 days, substantial improvement in lung CT imaging, as well as two consecutive negative RT-qPCR tests for viral RNA. Follow-up participants underwent examination in the fourth week after being discharged. For this longitudinal study, we classified the patients into three distinct groups: a mild group (n=17), a moderate group with abnormally high levels (AHL) of platelet counts (HMo, n=15), and a moderate group with Normal Levels (NMo, n=266) of platelet counts within the normal range (**Figure 1A**).

### Data collection and laboratory procedures

We collected extensive admission data by gathering various types of information, including electronic medical records, epidemiological data, demographic information, clinical details, laboratory results, treatment history, and outcome records. To ensure consistency and accuracy, we utilized a standardized data collection form based on the WHO/International Severe Acute Respiratory and Emerging Infection Consortium case record form for severe acute respiratory infections.

Samples of ethylenediaminetetraacetic acid-anticoagulated peripheral blood were obtained from patients who underwent COVID-19 testing either upon hospitalization or on the 30th day after discharge. These samples were then subjected to routine blood examinations, which involved analyzing the complete blood count. This comprehensive analysis included assessing and quantifying various components such as white blood cells, neutrophils and lymphocytes. To clarify the criteria used for the inclusion or exclusion of study participants, **Figure 1A** was provided. White blood cell differentials, including neutrophil and lymphocyte counts, and platelet counts were monitored, and data were collected from ninety-two healthy, unvaccinated individuals.

### Flow cytometric analysis

We obtained 2 mL of peripheral blood samples with ethylenediaminetetraacetic acid (EDTA) as an anticoagulant from patients diagnosed with COVID-19 before initiating treatment and 30 days after hospital discharge. These samples were used to analyze the phenotypic characteristics of lymphocytes. The measurement methods used were previously described (Jiang et al., 2020).

To perform the phenotypic characterization of lymphocytes, we utilized specific antibodies. The antibodies used included peridinin chlorophyll protein (PerCP)-conjugated anti-human CD3 monoclonal antibody (Ab), allophycocyanin (APC)-conjugated anti-human CD4 Ab, FITC-conjugated anti-CD127 Abs, PE-Cy7-conjugated anti-CD25 Ab, PE-conjugated anti-FoxP3 Ab, and APC-conjugated anti-human CD19 Ab from BD Biosciences (California, USA). Additionally, APC/Cy7-conjugated anti-human CD8 Ab, APC-conjugated anti-human CD16 Ab, as well as Brilliant Violet 510 (BV-510)-conjugated anti-human CD56 Ab were obtained from BioLegend (USA).

Flow cytometry analysis was conducted using the BD FACS Canto II, a multicolor flow cytometry system from BD Biosciences. The gating strategy for identifying CD4^+^ T cells, CD8^+^ T cells, DN T cells, Tregs, CD19^+^ B cells, and NK cells was defined as CD3^+^CD4^+^, CD3^+^CD8^+^, CD3^+^CD4^-^CD8^-^, CD4^+^CD25^+^CD127^low^/FOXP3^+^, CD3^-^CD19^+^, and CD3^-^CD16^+^/CD56^+^ respectively.

### Statistical analysis

The results were presented as medians (interquartile range, IQR). The D’Agostino & Pearson omnibus normality tests were utilized to compare the distributions. A power analysis conducted using the R package pwr for Groups HMo and NMo indicated a power of 0.85, based on the specified sample sizes and a significance level of 0.05. In instances where parameters were not measured in normal controls, we employed the usual clinical values of men and women from our hospital as reference ranges and compared the patient groups to these ranges. If the data did not meet the assumptions of parametric statistics, skewed quantitative data (such as counts of lymphocyte, B cell, T cell, CD4 and CD8 T cell, DN T cell, Tregs and NK cell) were assessed using a non-parametric Kruskal-Wallis multiple comparisons test. Categorical variables were compared using Chi-square tests or Fisher exact tests (for example, sex and age). Spearman correlation coefficients were computed to establish the relationship between platelet count and B cell counts. A p-value less than 0.05 was considered statistically significant. The data analysis was conducted using SPSS software (SPSS standard, version 25.0; SPSS, Inc., Chicago, IL, USA).

## Results

### Clinical evaluation of patients

Mild COVID-19 cases presented with minimal symptoms and no radiographic abnormalities. When compared to healthy unvaccinated controls (NC), the white blood cell, neutrophil, lymphocyte, and platelet counts remained within normal ranges in both the NC and mild groups upon admission and at DPSO 48 (**Table 1**). This indicates the absence of thrombocytopenia and lymphopenia in mild cases, making this group a suitable control for the analysis of moderate cases (NMo and HMo groups).

**Table 1:** White blood cell differentials and platelet counts in mild COVID-19 cases (Mild) on admission and DPSO48 comparable to normal controls (NC).

	NC (n=92)	Mild (n=17)		p value
Gender (male/female)	73/19	14/3		NS
Age (year)	24.5 (22.0, 27.0)	40.5 (26.0, 49.5)		0.0003
BMI (Kg/m^2^)	22.30 (20.30, 25.00)	22.42 (19.16,23.97)		NS
		Admission	DPSO50	
WBC counts (×10^9^/L)	5.77 (5.06,6.83)	5.42 (4.02,7.66)	5.52 (5.03,7.57)	
>10 (×10^9^/L)	3/92 (3.3)	0/17 (0)	1/7 (14.3)	NS
Neutrophil counts (×10^9^/L)	3.26 (2.70,4.17)	3.00 (2.16,5.2)	2.90 (2.45,4.40)	
>6.3 (×10^9^/L)	4/92 (4.4)	0/17 (0)	0/7 (0)	NS
Lymphocyte counts (×10^9^/L)	1.82 (1.49,2.23)	1.40 (1.08,2.15)	2.00 (1.80,2.32)	
< 1.0 (×10^9^/L)	0/92 (0)	1/17 (5.9)	0/7 (0)	NS
Platelet counts (×10^9^/L)	243.5 (219.0,268.0)	192.0 (168.0,218.0)	199.0 (182.0,251.0)	
< 100 (×10^9^/L)	0/82 (0)	0/17 (0)	0/7 (0)	NS

Data presented as median (interquartile range, IQR). p values were determined using the Mann-Whitney U test or Fisher’s exact test, as applicable. NS denotes non-significant findings.

Among the 298 patients, there were 155 males and 143 females, with a median age of 46 (IQR 36–54) years. There is a higher male-to-female patient ratio in the mild group compared to the moderate cases (p = 0.011). The patients in the mild group are younger than those in the moderate group (p = 0.03), with a median age of 41 (IQR 26-50) years for the mild group and 46 (IQR 36-54) years for the moderate group (**Table 1**).

A total of 102 (34.2%) out of the 298 patients had concurrent medical conditions, with hypertension being the most prevalent, followed by diabetes, chronic liver disease, chronic lung disease, and chronic heart disease, HBV and chronic renal disease. In moderate COVID-19 patients on DPSO 48, there were a subgroup of cases (5.3%) with high platelet levels above the nationally accepted threshold (AHL > 300×10^9^/L) (**Figure 1B**), and they were classified as the High-Platelet moderate (HMo) cohort. Three groups of COVID-19 patients, categorized as mild, HMo and NMo, were followed up for approximately 48.0 (IQR 43.0-53.0) days after the onset of symptoms (**Table 2**).

**Table 2:** Demographics and comorbidities of cases and symptom profile analysis.

	All(N=298)	Mild(N=17)	Moderate(N=281)	
**Variables**				**P**
Gender(male/female)	155/143	14/3	141/140	0.01
Age(median,range)	46(36,54)	41(26,50)	46(36,54)	0.03
Body temperature	37.0(36.5,37.5)	36.8(36.6,37.3)	37.0(36.5,37.5)	0.97
Systolic pressure (mmHg)	128.0(119.8,140.0)	135.0(120.5,144.5)	128.0(119.5,140.0)	0.54
Diastolic pressure (mmHg)	82.0(75.0,90.0)	82.0(76.5,96.5)	82.0(75.0,90.0)	0.34
**Pre-existing disorders (yes/ no)**				
Chronic heart disease	4/294	0/17	4/277	1.0
Diabetes	26/272	2/15	24/257	0.65
Hypertention	48/250	2/15	46/235	1.0
Chronic renal disease	1/297	0/17	1/280	1.0
Cancer	0/298	0/17	0/281	1.0
Chronic liver disease	14/284	1/16	13/268	0.57
HBV	1/297	0/17	1/280	1.0
Chronic lung disease	8/290	0/17	8/273	1.0
**Symptoms(yes/no)**				
Fever	193/105	8/9	185/96	0.12
Dry cough	119/179	7/10	112/169	1.0
Fatigue	94/204	6/11	88/193	0.79
Sore throat	44/254	3/14	41/240	0.72
Runny nose	26/272	3/14	23/258	0.18
Sputum production	157/141	6/11	151/130	0.21
Dizzy or headache	29/269	0/17	29/252	0.39
Nausea or vomiting	29/269	0/17	29/252	0.39
Myalgia	24/274	2/15	22/259	0.64
Poor apprtite	16/282	0/17	16/265	0.61
Diarrhea	57/241	3/14	54/227	1.0
**Post-symptom onset (days)**	48.0(43.0,53.0)	48.0(40.5,56.5)	48.0(43.0,53.0)	0.91

Results are presented as median and interquartile range (IQR). *P* values are the results of Pearson χ^2^ tests or Fisher’s exact test for categorical variables.

Restorations of infectious inflammation parameters and dynamic changes of cell counts of platelet and lymphocyte, and platelet-lymphocyte ratio in the recovery phase of COVID-19.

### Inflammatory responses and lymphocytopenia were fully resolved in COVID-19 patients during the convalescent period

**Figure 2:**
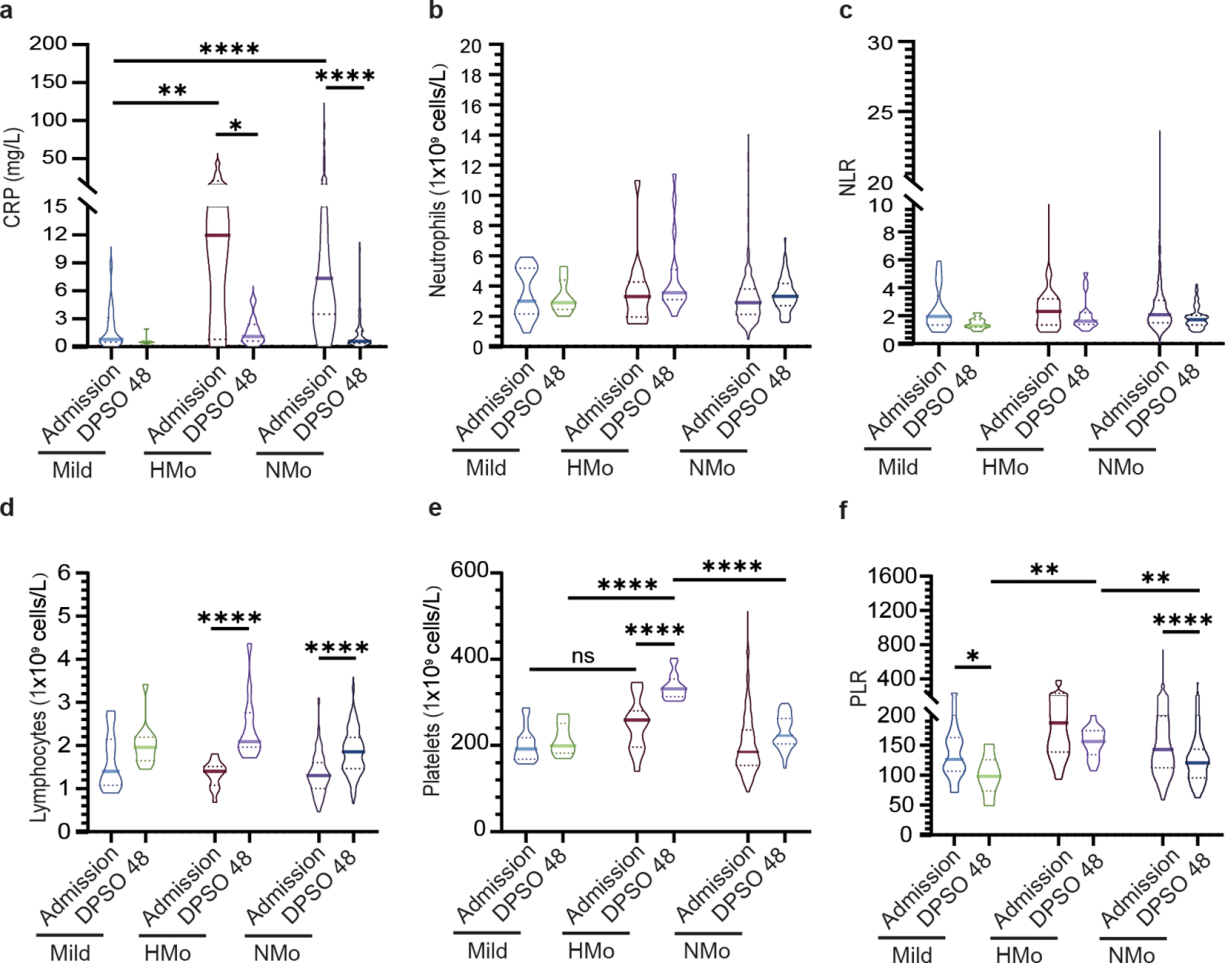
Dynamic profiles of inflammatory, platelet, immune-cells, and cellular ratios among different groups at admission and DPSO48. **(A)** The concentration of C-reactive protein (CRP) levels significantly increased in moderate cases, and subsequently normalized during the recovery phase. **(B, C)** Dynamic changes in white blood cells (WBC) and neutrophil to lymphocyte ratio (NLR). **(D)** The lymphocyte count increased during the recovery period in both HMo and NMo groups. **(E, F)** Dynamic changes in platelets and platelets to lymphocyte ratio (PLR). P values were calculated using non-parametric Kruskal-Wallis multiple comparisons test. Statistical significance is denoted by *p<0.05, **p<0.01, and ****p<0.0001. ns, no significant. DPSO 48, 48 Days post-symptom onset. NMo, moderate cases with platelet counts in the normal range. HMo, moderate cases with high platelet counts (>300×10^9^/L).

As shown in **Table 3**, CRP concentrations were low in mild cases during the acute phase, remaining similar in the convalescent phase. In contrast, moderate cases exhibited significantly elevated CRP levels upon admission, which normalized by day 48 post-symptom onset (DPSO), with no significant differences between mild and moderate groups (**Figure 2A**). There were no statistically significant differences in neutrophil counts or the neutrophil-to-lymphocyte ratio (NLR) between the mild and moderate groups during both the acute and convalescent stages (**Figures 2B, C**). Notably, lymphocytopenia, characterized by reduced lymphocyte counts in the acute phase, was fully resolved in moderate cases during the convalescent period (**Figure 2D**).

**Table 3:** Dynamic alterations in inflammatory, lymphocyte-subsets indicators of cases from admission to DPSO48.

		Mild(N=17)			Moderate(N=281)			
		On admission	DPSO 48		On admission	DPSO 48		
Variables	Reference ranges			P			P	*P
CRP, mg/L	≤10	0.80(0.50,3.11)	0.50(0.46,0.50)	0.07	7.68(3.50,21.68)	0.60(0.48,1.70)	<0.0001	0.27
WBC count, ×10^9^ /L	4-10	5.42(4.02,7.66)	5.52(5.03,7.57)	0.47	4.72(3.70,6.00)	6.01(5.11,6.90)	<0.0001	0.84
Neutrophil count, ×10^9^ /L	1.8-6.3	3.00(2.16,5.20)	2.90(2.45,4.40)	0.95	2.90(2.13,3.80)	3.36(2.73,4.22)	0.001	0.44
Lymphocytes, ×10^9^ /L	1.1–3.2	1.40(1.08,2.15)	2.00(1.80,2.32)	0.05	1.30(1.00,1.60)	2.00(1.69,2.37)	<0.0001	0.49
NLR		1.96(1.35,3.41)	1.30(1.20,1.72)	0.18	2.10(1.51,3.10)	1.69(1.37,2.04)	0.0002	0.17
Platelets, ×10^9^ /L	100-300	192.0(168.0,218.0)	199.0(182.0,251.0)	0.36	188.5(155.3,241.8)	246.0(207.0,289.0)	<0.0001	0.07
PLR		126.4(106.6,162.8)	97.8(73.3,125.5)	0.047	144.7(113.4,201.2)	124.9(98.9,154.1)	<0.0001	0.06
B cell, cells/μL	90–560	266.4(121.0,480.0)	232.2(169.8,301.2)	0.64	151.0(99.0,223.0)	185.6(138.9,254.3)	0.007	0.16
B cell %		14.02(8.64,32.78)	13.05(9.45,15.65)	0.41	11.64(8.95,20.08)	11.1.0(8.05,14.35)	0.06	0.19
CD4 T cell, cells /μL	414-1123	529.5(439.3,770.8)	724.8(585.0,814.9)	0.14	546.4(389.0,747.5)	657.8(510.1,830.0)	0.0003	0.48
CD4 T cell %		35.65(33.51,46.77)	37.00(32.70,40.60)	0.52	41.69(32.86,49.82)	36.80(32.05,42.15)	<0.0001	0.71
CD8 T cell, cells /μL	238-874	398.0(288.5,527.0)	434.1(406.2,669.1)	0.21	326.0(232.0,457.8)	474.2(370.9,609.1)	<0.0001	0.76
CD8 T cell %		27.12(21.45,37.44)	26.00(23.60,28.80)	0.83	24.00(17.03,32.73)	27.10(22.75,31.70)	0.07	0.58
CD4 / CD8 T cell ratio		1.49(1.12,1.61)	1.40(1.20,1.90)	0.84	1.69(1.14,2.49)	1.37(1.10,1.68)	0.0005	0.58
NK cell, cells /μL	150–1100	315.0(166.5,391.0)	279.3(123.5,475.4)	0.96	217.0(142.3,385.5)	296.5(172.6,450.3)	0.049	0.74
NK cell %		27.93(9.06,35.00)	16.50(10.20,25.93)	0.36	16.49(11.80,29.00)	18.20(12.40,25.60)	0.94	0.53

The data are presented as median and interquartile range (IQR). P values are the results of Mann-Whitney U test for continuous variables. *P indicates whether there is a statistically significant or non-statistically significant difference between mild and moderate cases on DPSO 48. DPSO 48, 48 Days post-symptom onset. CRP, C-reactive protein; WBC, White blood cell; NLR, neutrophil to lymphocyte ratio; PLR, platelet-to-lymphocyte count ratios; NK cells, natural killer cells.

### 5.3% of moderate cases exhibited high platelet counts

In DPSO 48, the median platelet counts in mild cases were 199.0 (IQR 182.0-251.0) cells/L, while in moderate cases, the median platelet counts were 246.0 (IQR 207.0-289.0) cells/L, representing a 23.6% higher count. However, this difference did not reach statistical significance (p = 0.068). Interestingly, on DPSO 48, 5.3% of moderate cases exhibited platelet counts that surpassed the high threshold level. This was in contrast to their admission levels, as well as the levels observed in both the mild and NMo groups on DPSO 48 (**Figure 2E**). Higher platelet-to-lymphocyte count ratios (PLR) on admission, primarily driven by lymphopenia, are associated with elevated morbidity and mortality in COVID-19 patients (Sarkar et al., 2022). During the recovery phase, while lymphocyte counts returned to the normal range, the PLRs were lower in mild and NMo cohorts at DPSO 48. In HMo cases, both lymphocyte and platelet counts showed a significant increase, and the PLR at DPSO 48 remained unchanged compared to the ratio observed upon admission (**Figure 2F**).

### Higher counts of total T cells, CD4 and CD8 T cells, and Tregs counts in HMo cases with high platelet counts during convalescence

**Figure 3:**
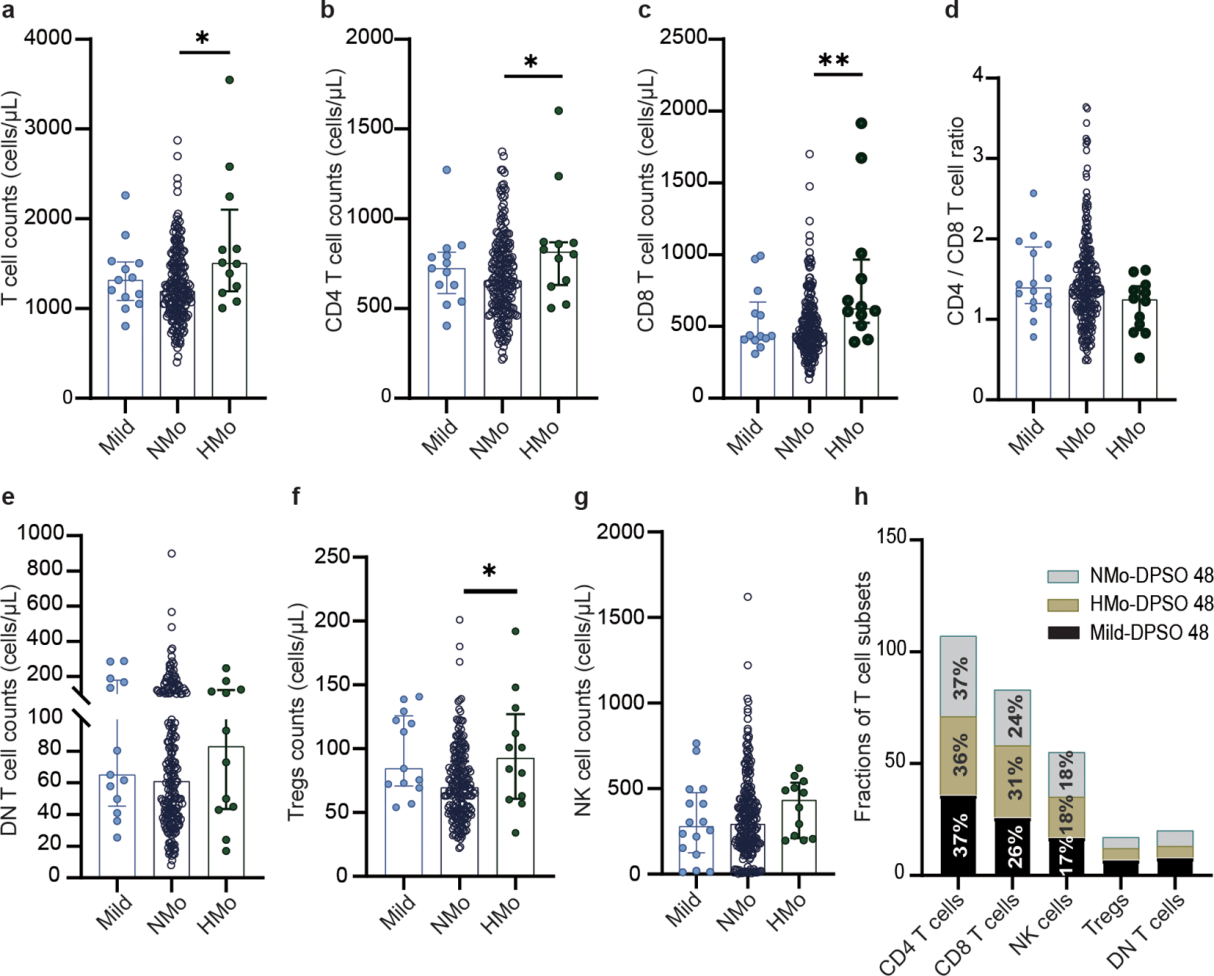
The differences of the counts of total T cell and their subsets counts, NK cell counts, and frequencies of T cell subsets among mild, Nmo and Hmo cases during convalescence. Compared the changes in the number of **(A)** total T cells, **(B)** CD4 T cells, **(C)** CD8 T cells, **(D)** CD4/CD8 T cell, **(E)** DN T cells, **(F)** Tregs and **(G)** NK cells counts at DPSO 48 among the mild, NMO and HMo group. **(H)** The fractions of T cell subsets in each group at DPSO 48. DPSO 48, Days post-symptom onset. NMo, moderate cases with platelet counts in the normal range. HMo, moderate cases with high platelet counts (>300×10^9^/L). P values were calculated using non-parametric Kruskal-Wallis multiple comparisons test. Statistical significance is denoted by *p<0.05, **p<0.01.

As shown in **Table 4**, individuals with high platelet counts on DPSO 48 exhibited a higher total T cell count (26.4% increase) compared to those with normal platelet counts (NMo), with median counts for NMo and HMo being 1191.0 (IQR 964.6-1484.0) and 1505.0 (IQR 1192.0-2103.0) cells/µL, respectively. Similarly, the CD4 T cell count was 24.4% higher in HMo compared to NMo, with median counts of 655.6 (IQR 503.1-817.8) and 815.3 (IQR 631.0-869.7) cells/µL, respectively. Moreover, the CD8 T cell count was 36.9% higher in HMo than in NMo, with median counts of 624.2 (IQR 525.1-964.9) and 456.1 (IQR 367.5-595.7) cells/µL, respectively. Additionally, the Tregs count in HMo was 33.9% higher than in NMo, with median counts of 92.11 (IQR 60.96-126.60) and 68.78 (IQR 50.72-89.84) cells/µL, respectively (**Figures 3A–C, F**).

**Table 4:** Lymphocyte and its subtype cell profiles in convalescent patients with or without high platelet counts in moderate COVID-19.

	NMo(N=266)	HMo(N=15)	
Variables			P
CRP, mg/L	0.58(0.40,1.70)	0.94(0.56,1.88)	0.34
WBC count, ×10^9^ /L	5.92(5.03,6.85)	6.30(5.40,8.04)	0.11
Neutrophil count, ×10^9^ /L	3.33(2.70,4.18)	3.40(3.10,4.90)	0.28
Lymphocytes, ×10^9^ /L	2.00(1.61,2.37)	2.20(1.91,2.40)	0.12
NLR	1.72(1.35,2.03)	1.58(1.37,2.23)	0.92
Platelets, ×10^9^ /L	222.5(203.5,262.8)	331.0(313.0,359.0)	<0.0001
PLR	119.1(94.4,144.1)	158.6(139.1,174.2)	0.0006
B cell, cells/μL	185.2(138.9,250.9)	229.1(126.2,418.9)	0.34
B cell %	11.10(8.15,14.35)	10.30(6.93,14.65)	0.56
Total T cell, cells /μL	1191.0(964.6,1484.0)	1505.0(1192.0,2103.0)	0.01
T cell %	68.7(62.0,75.5)	64.4(62.6,79.9)	0.58
CD4 T cell, cells /μL	655.6(503.1,817.8)	815.3(631.0,869.7)	0.04
CD4 T cell %	36.80(32.10,42.15)	36.10(27.33,41.98)	0.55
CD8 T cell, cells /μL	456.1(367.5,595.7)	624.2(525.1,964.9)	0.003
CD8 T cell %	27.00(22.60,31.50)	30.75(25.95,35.68)	0.08
CD4 / CD8 T cell ratio	1.38(1.11,1.70)	1.25(0.87,1.41)	0.07
NK cell, cells /μL	292.9(166.2,437.9)	432.6(210.6,533.6)	0.14
NK cell %	18.20(12.70,25.40)	17.60(9.13,26.95)	0.49
DN T cell, cells /μL	60.46(38.52,104.60)	82.96(43.38,122.70)	0.51
DN T %	5.10(3.70,8.78)	4.15(2.23,8.28)	0.25
Tregs, cells /μL	68.78(50.72,89.84)	92.11(60.96,126.60)	0.04
Tregs %	5.86(5.03,6.85)	5.64(4.65,8.11)	0.85

The data are presented as median and interquartile range (IQR). P values are the results of Mann-Whitney U test for continuous variables. NMo, moderate cases with platelet counts in the normal range. HMo, moderate cases with high platelet counts (>300×10^9^/L). CRP, C-reactive protein; WBC, White blood cell; NLR, neutrophil to lymphocyte ratio; PLR, platelet-to-lymphocyte count ratios; NK cells, natural killer cells; DN T, double negative T cells; Tregs, regulatory T cells.

On DPSO 48, there were no significant differences in the CD4/CD8 T cell ratio among HMo, NMo, and mild cases (**Figure 3D**). This could be attributed to the proportional increase in both CD4 and CD8 T cell counts. No significant variances were observed in the counts of DN T cells and NK cells among HMo, NMo, and mild cases (**Figures 3E, G**). Similarly, there were no significant disparities in the frequencies of T cell subsets (including CD4 and CD8 T cells, Tregs, DN T cells, and NK cells) among the HMo, NMo, and mild groups on DPSO 48 (**Figure 3H**, **Table 3**).

### B-cell counts and the correlation between platelet counts and B-cell counts during the convalescent period

**Figure 4:**
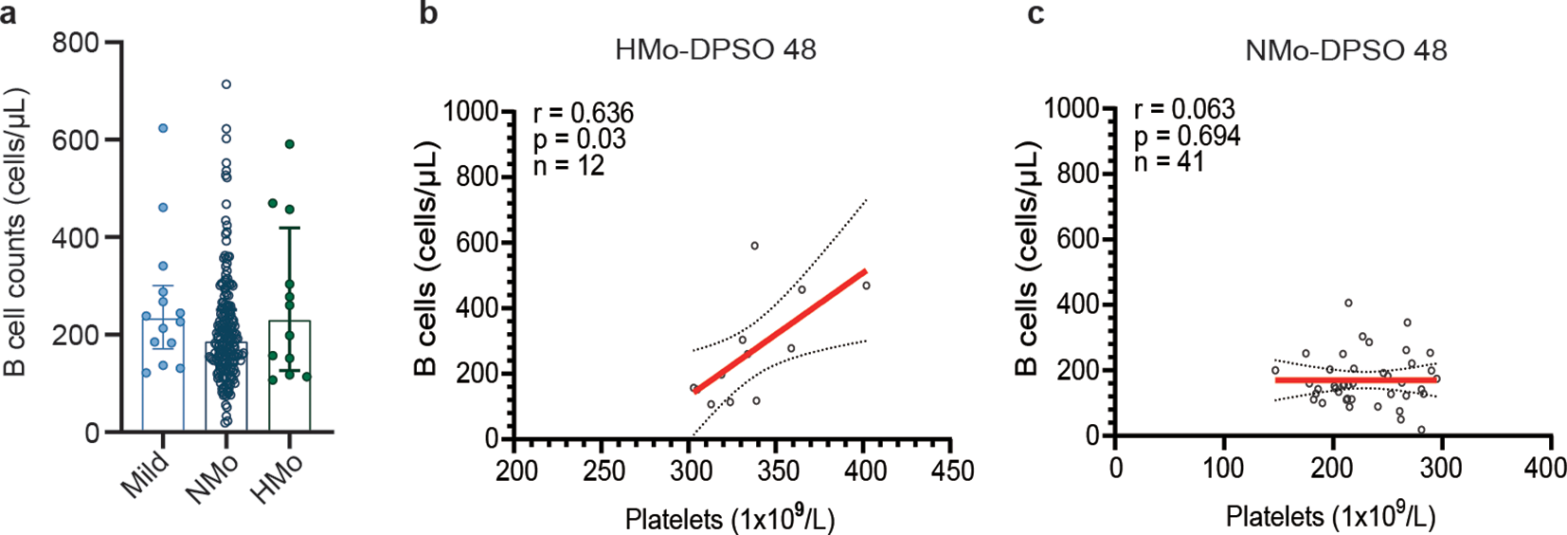
Relationships between platelet counts and B-cell counts during the convalescent period. **(A)** B cell counts showed no significant differences among the mild, NMo and HMo groups during the convalescence period. **(B)** Strong positive correlation was found between platelet counts and B cell counts in HMo cases. **(C)** No correlation was observed between platelet counts and B cell counts in NMo cases. The correlation was analyzed using Spearman’s rank correlation coefficients. DPSO 48, 48 Days post-symptom onset. NMo, moderate cases with platelet counts in the normal range. HMo, moderate cases with high platelet counts (>300×10^9^/L).

The median B cell counts in NMo cases were 185.2 (IQR 138.9-250.9) cells/µL, while in HMo cases the median B cell counts were 229.1 (IQR 126.2-418.9) cells/µL, representing a 23.7% higher count. However, this difference did not reach statistical significance (p > 0.05) (**Figure 4A**). A strong positive correlation was found between platelet counts and B cell counts in HMo cases (r = 0.636, p = 0.03, **Figure 4B**). Conversely, no correlation was observed between platelet counts and B cell counts in NMo cases (**Figure 4C**).

## Discussion

Lymphopenia and thrombocytopenia have been recognized as detrimental factors linked to unfavorable outcomes in the acute phase of SARS-COV-2 infection (Henry et al., 2020; Huang and Pranata, 2020; Lippi et al., 2020). Nevertheless, the exact mechanism connecting the abnormal correlation between lymphopenia and thrombocytopenia and their consequences to the disease necessitates further investigation.

During the recovery phase, a majority of COVID-19 patients generally experience a normalization of lymphocyte and platelet counts, as demonstrated by previous research (Mandal et al., 2021; Alfadda et al., 2022; Lechuga et al., 2023). In our investigation, we found that 5.3% of 281 moderate COVID-19 patients displayed elevated platelet counts (>300×10^9^/L) on DPSO 48, which aligns with the results reported in a previous longitudinal study spanning 7 months. In the aforementioned study, asymptomatic patients had a platelet count of 266.16 ± 80.6 (×10^9^/L, mean ± SD) after six months of follow-up, while symptomatic patients had a platelet count of 253.68 ± 52.2 (×10^9^/L, mean ± SD). By examining the platelet mean values and standard deviations reported in their findings (Alfadda et al., 2022), it suggests that there is a subgroup within their cohort whose platelet counts remained above 300×10^9^ cells/L even at the six-month follow-up.

Interestingly, our study found that convalescent COVID-19 patients with elevated platelet counts had higher cell counts of total T cells, CD4 and CD8 T cells, and Tregs compared to convalescent patients with normal platelet counts. Previous reports have not specifically examined the relationship between platelets and T cell subsets in convalescent COVID-19 patients. Therefore, our study provides initial evidence showing an association between higher platelet counts and increased cell counts of total T cells and its subsets in convalescent COVID-19 patients.

Numerous pieces of evidence indicate that platelets have the ability to influence the functions of CD4 and CD8 T cells (Morrell et al., 2014; Yu et al., 2020). However, our understanding of their role in the expansion or proliferation of these T cells remains limited. A study conducted by Elzey et al. revealed that a reduction in platelet levels led to a decrease in the production of CD8 T cells (Mandal et al., 2021). Conversely, when platelets were reintroduced in platelet-depleted mice, the number of CD8 T cells in the spleen and liver increased after an infection with lymphocytic choriomeningitis virus (Mandal et al., 2021). These findings emphasize the significance of platelets in the expansion of antigen-specific CD8 T cells. As for CD4 T cells, mitochondria derived from platelets were found to directly and differentially up-regulate Naïve and central memory CD4 T cells, while down-regulating effector memory CD4 T cells (Morrell et al., 2014).

Recently, there has been a suggestion that platelet-derived 5-HT may be involved in the underlying mechanism of LC (Wong et al., 2023). Previous studies have demonstrated that 5-HT promotes the proliferation of both B cells and T cells (Aune et al., 1993; Iken et al., 1995; Abdouh et al., 2001). In our study, although there was no significant difference in B cell counts between HMo and NMo cases, we observed a positive correlation between platelet counts and B cell counts only in HMo cases (r = 0.636, p = 0.03). This suggests that higher platelet counts are associated with higher B cell counts in a subset convalescent COVID-19 patients.

Platelets exert harmful effects primarily through the inflammatory response during viral infection (Morrell et al., 2014). Inflammatory-activated platelets enhance the adhesion of lymphocytes to the endothelium, directing them towards endothelial veins in various inflammatory sites and causing damage (Koupenova et al., 2018). In the convalescent phase of COVID-19, the inflammatory responses subside, as indicated by low levels of CRP, an inflammatory marker, in both HMo and NMo cases in this study. Therefore, during the convalescence of COVID-19, platelets mainly play a role in modulating immune homeostasis in patients. Persistent SARS-CoV-2 shedding is strongly linked to reduced T cell and B cell counts, which can contribute to chronic immune activation in LC (Hu et al., 2020).

The mechanisms linking elevated platelet counts to increased T-cell numbers remain unclear. In addition to the previously mentioned role of platelet-derived 5-HT in promoting T-cell proliferation, the convalescent phase of COVID-19 may involve diminished inflammatory cytokines that also connect elevated platelets and T-cell counts. For instance, high mobility group box 1 (HMGB1), which is actively secreted by necrotic cells in response to inflammatory signaling, may contribute to the hyper-inflammatory response seen in COVID-19 (Al-Kuraishy et al., 2022), leading to complications such as acute respiratory distress syndrome (ARDS) and thrombosis. Notably, plasma levels of HMGB1 show a negative correlation with platelet counts in immune thrombocytopenia (ITP). The natural HMGB1 inhibitor, 18β-glycyrrhetinic acid (18β-GA), exhibits anti-inflammatory and immune-modulatory effects. It enhances the production of Tregs, restores the balance of CD4 T-cell subsets, and increases the suppressive functions of Tregs by inhibiting HMGB1 activity in ITP patients. These findings suggest that inhibiting HMGB1 signaling may effectively restore immune balance in ITP (Wang et al., 2023). Moreover, our study indicates a correlation between elevated platelet counts and Tregs, CD4, and CD8 T cells, without altering the CD4/CD8 T-cell ratio, implying a restoration of immune homeostasis. This is further supported by our observation that the frequencies of all T-cell subsets remained consistent among healthy controls, mildly ill patients, and those with mild COVID-19 on day 48 post-symptom onset (DPSO). In contrast, individuals with long COVID showed increased frequencies of CD8 T cells, Tregs, and natural killer (NK) cells compared to recovered patients, highlighting disruptions in immune homeostasis and a persistent cytotoxic memory immune response (Galan et al., 2022).

Tregs play a crucial role in autoimmune diseases. Clinical studies have revealed that COVID-19 patients exhibit lower levels of Tregs in comparison to the general population. This reduction in Tregs can result in compromised control over inflammation, an imbalance in the Treg/Th17 ratio, and an increased susceptibility to respiratory failure (Alsalman et al., 2022). Additionally, decreased Tregs contribute to the development of LC and exacerbate the prognosis of the disease (Dhawan et al., 2023). Emerging evidence has substantiated the crucial role of Tregs in maintaining immune balance and preventing the onset of autoimmune disorders. Furthermore, defects or deficiencies in Tregs have been correlated with a wide array of autoimmune conditions, allergies, and chronic inflammatory diseases (Cheru et al., 2023). Our study reveals a positive correlation between elevated platelet counts and increased Tregs levels, suggesting that platelets and/or their mediators may stimulate the expansion of Tregs. Given the significant role of Tregs in the immune response, further investigation is warranted to explore the connections between platelets and Tregs in the context of LC.

CD8 T cells have been shown to be a crucial type of immune cell induced by vaccination for protection against SARS-CoV-2. Activation and proliferation of CD8 T cells targeting the spike protein of SARS-CoV-2 can be observed following breakthrough infections. Moreover, this response is only observed in individuals who were previously vaccinated, while it is absent in unvaccinated individuals (Painter et al., 2023). These findings are consistent with the results of Zhang et al.’s study, which identified CD8 T cells specific to two subgroups of the vaccine and potentially serving as long-term memory cells (Zhang et al., 2023). In our study, we found that individuals with higher platelet counts have higher CD8 T cell counts (by 36.9%) on DPSO 48, suggesting that platelets may play a key role in enabling T cells to provide effective long-term protection against future SARS-CoV-2 infections.

In this study, we acknowledge several limitations that may affect the interpretation of our findings. The generalizability of our results is constrained by the study population and context, and we did not assess SARS-CoV-2-specific immune responses, which could have offered deeper insights into the immune dynamics involved. Additionally, potential confounding factors may influence our results, necessitating careful consideration in future analyses. The absence of a healthy control group is another notable limitation that we plan to address in upcoming studies to enhance the robustness of our conclusions. Furthermore, we encountered significant limitations related to the short duration of follow-up, particularly for patients affected by long COVID (LC). To fully examine variations attributed to disease severity, treatment, and other potential factors, large-scale studies with extended follow-up periods are essential. While our study provides valuable insights into lymphocyte subtype cell profiles in post-COVID-19 individuals, employing additional analytical methodologies focused on immune cell subtypes will yield a more detailed assessment of immune dysregulation in recovering patients and the specific cellular characteristics associated with LC.

In summary, 5.3% of moderate COVID-19 unvaccinated patients show high platelet counts (> 300x10^9^/L), which is linked to increased levels of total T-cells, CD4 and CD8 T-cells, and Tregs compared to patients with normal platelet counts. This does not affect the CD4/CD8 T-cell ratio. Furthermore, the significant positive correlation observed between platelet counts and B cell counts, particularly in cases of hematological malignancies, provides compelling evidence of a strong association and suggests underlying pathways linking platelets and B cells. These findings are crucial for advancing our understanding of platelet-related immunopathology and may help identify reliable biomarkers for recognizing patients with immune disorders who have achieved immunological balance during their recovery.

## Data Availability

The raw data supporting the conclusions of this article will be made available by the authors, without undue reservation.
